# Comprehensive analyses identify RIPOR2 as a genomic instability-associated immune prognostic biomarker in cervical cancer

**DOI:** 10.3389/fimmu.2022.930488

**Published:** 2022-08-26

**Authors:** Fangfang Xu, Chang Zou, Yueqing Gao, Jiacheng Shen, Tingwei Liu, Qizhi He, Shuangdi Li, Shaohua Xu

**Affiliations:** ^1^ Department of Gynecology, Shanghai First Maternity and Infant Hospital, School of Medicine, Tongji University, Shanghai, China; ^2^ Department of Pathology, Shanghai First Maternity and Infant Hospital, School of Medicine, Tongji University, Shanghai, China

**Keywords:** RIPOR2, cervical cancer, genomic instability, tumor microenvironment, prognostic model

## Abstract

Cervical cancer (CC) is a malignancy that tends to have a poor prognosis when detected at an advanced stage; however, there are few studies on the early detection of CC at the genetic level. The tumor microenvironment (TME) and genomic instability (GI) greatly affect the survival of tumor patients *via* effects on carcinogenesis, tumor growth, and resistance. It is necessary to identify biomarkers simultaneously correlated with components of the TME and with GI, as these could predict the survival of patients and the efficacy of immunotherapy. In this study, we extracted somatic mutational data and transcriptome information of CC cases from The Cancer Genome Atlas, and the GSE44001 dataset from the Gene Expression Omnibus database was downloaded for external verification. Stromal components differed most between genomic unstable and genomic stable groups. Differentially expressed genes were screened out on the basis of GI and StromalScore, using somatic mutation information and ESTIMATE methods. We obtained the intersection of GI- and StromalScore-related genes and used them to establish a four-gene signature comprising RIPOR2, CCL22, PAMR1, and FBN1 for prognostic prediction. We described immunogenomic characteristics using this risk model, with methods including CIBERSORT, gene set enrichment analysis (GSEA), and single-sample GSEA. We further explored the protective factor RIPOR2, which has a close relationship with ImmuneScore. A series of *in vitro* experiments, including immunohistochemistry, immunofluorescence, quantitative reverse transcription PCR, transwell assay, CCK8 assay, EdU assay, cell cycle detection, colony formation assay, and Western blotting were performed to validate RIPOR2 as an anti-tumor signature. Combined with integrative bioinformatic analyses, these experiments showed a strong relationship between RIPOR2 with tumor mutation burden, expression of genes related to DNA damage response (especially PARP1), TME-related scores, activation of immune checkpoint activation, and efficacy of immunotherapy. To summarize, RIPOR2 was successfully identified through comprehensive analyses of the TME and GI as a potential biomarker for forecasting the prognosis and immunotherapy response, which could guide clinical strategies for the treatment of CC patients.

## Introduction

Cervical cancer (CC) is the fourth deadliest malignancy in women worldwide. In 2018, there were about 569,000 confirmed cases of CC and about 311,000 deaths worldwide (1). Approximately 85% of deaths from CC occur in underdeveloped countries and areas (2), with the highest mortality rates found in parts of America and sub-Saharan Africa (3).

In addition to the high morbidity and poor prognosis of CC, the refractoriness of the disease tends to increase with the stage of tumor development at diagnosis. Around two-thirds of this population have developed locally advanced cervical cancer by the time anti-tumor treatment is initiated. Even when these type of CC patients are operated with proper multidisciplinary treatment, they still always receive low survival rates. The 3-year local control rates for patients diagnosed with early-stage and advanced CC are 87%–95% and 74%–85%, respectively (4, 5).

Genomic instability (GI) has long been considered a contributing factor to tumorigenesis and acquisition of resistance to therapy. On the one hand, GI is a “facilitating characteristic” of various tumor types and encourages the expression of hallmarks (6). Simultaneously, tumor patients with higher levels of GI potentially present more immunogens compared with those with a lower genetic burden (7). Therefore, GI may have potential applications both as a prognostic marker in cancer and as a source of novel drug targets. Moreover, GI may be associated with the tumor microenvironment (TME). Distinguishing features of the TME include vascular abnormalities, hypoxia, and cell constituents including cancer cells, immune-related cells (such as T cells, dendritic cells, and fibroblasts), as well as extracellular matrix and blood vessels (8). The TME can downregulate GI by suppressing processes related to the DNA damage response (DDR) (9). Hypoxia plays an essential part in this process by repressing c-MYC, which accelerates proliferation-related transcription of mismatch repair (MMR) promoters Mlh1 and Mlh2, thereby indirectly downregulating the expression of Mlh1 and Mlh2 (10). The rate of binding of suppressive transcriptional factors MNT and MAD1 to promoters of Mlh1 and Mlh2 also increases to inhibit the expression of MMR genes. Hypoxia also participates in the activation of the BRCA1 and RAD51 genes (11, 12), which can further increase GI.

In this study, we observed significant differences in the proportions of stromal-cell components, as calculated by the ESTIMATE algorithm, between patient groups with large differences in the number of mutations. We established a four-gene prognostic prediction model by least absolute shrinkage and selection operator (12) Cox analysis, based on the intersection of StromalScore-related and GI-related genes (GIRGs). Combined with multiple prognostic factors, this comprehensive predictor was validated as robust and effective in the external dataset GSE44001. After successfully constructing and validating the prognostic model, we calculated risk scores for CC samples and divided them into two groups based on these scores, and found that the two groups differed significantly with respect to overall survival (OS) rate. We compared immune- and genomic-related features including distribution of common immune checkpoints (ICPs), correlations with DDR genes, and immune cell distributions between different risk groups using various tools—maftools, the CIBERSORT algorithm, single-sample gene set enrichment analysis (ssGSEA), etc. Among the genes used to construct the model, only Rho family-interacting-cell polarization regulator 2 (RIPOR2) was closely related to ImmuneScore; therefore, RIPOR2 was chosen as a hub gene for subsequent analyses. Generally, the high RIPOR2 expression group had a lower tumor mutation burden (TMB), higher ESTIMATEScore, and higher proportions of ICPs, and patients in this group responded better to immunotherapy with PD-1 alone or combined with CTLA4. Notably, high RIPOR2 expression was associated with significantly higher levels of CD8 T cells. For validation, we overexpressed RIPOR2 in SiHa and HeLa cell lines and carried out a series of experiments to verify the important role of RIPOR2 in CC, including immunohistochemistry (IHC), immunofluorescence (IF), quantitative reverse transcription PCR (RT-qPCR), transwell assays, cell counting kit-8 (CCK8) assays, EdU assay, cell cycle detection, colony formation assay, and Western blotting (WB). The RT-qPCR and WB results demonstrated that the distribution of ICPs and DDR-related genes differed among CC cell lines with differential RIPOR2 expression. Importantly, poly (ADP-ribose) polymerase 1 (PARP1) was found to have a strong relationship with RIPOR2; this finding could guide clinical therapeutic strategies used in CC patients. Other experimental results showed that RIPOR2 represented an anti-tumor signature in CC, consistent with the results of the bioinformatical analyses. Comparisons based on GSEA were also carried out; pathways related to mediating fluid immunity and cellular immunity were significantly enriched in the high RIPOR2 expression cohort. Moreover, patients with low RIPOR2 expression tend to have a “desert” immune phenotype, which may make them less likely to benefit from immunotherapy. This study identified a prognostic biomarker based principally on GI and TME, thereby offering new biomolecular targets for clinical therapy for CC.

## Materials and methods

### Data collection

Somatic mutational spectra and transcriptome data of 306 CC tumor cases were obtained from The Cancer Genome Atlas (TCGA; https://portal.gdc.cancer.gov/), and clinical data were extracted from cBioportal (http://www.cbioportal.org), to construct a prognostic model for CC. The expression matrix and clinical characteristics of the GSE44001 dataset (300 CC samples) were downloaded from the Gene Expression Omnibus (GEO) database (https://www.ncbi.nlm.nih.gov/geo/) (13).

### Group division and other analyses based on mutation data

The somatic mutational information of CC cases from the TCGA was stored in MAF form. According to the somatic mutation profiles, we calculated the cumulative number of somatic mutations per sample, then classified all the patients in the highest and lowest quartiles into two mutator phenotypes, denoted genomic unstable (GU) and genome stable (14), respectively. The top 20 genes with the highest cumulative number of mutations in the GU and GS groups are shown in descending order separately. The mutation score of each sample (TMB) was calculated using the following formula: (total mutation/total covered bases) × 10^6^. The mutation information of genes was compared between the GU and GS groups and between the different risk groups as determined by the prognostic model, and genes with co-mutations were also identified *via* the maftools package (15).

### Extraction of genomic instability-related genes and stromal-related key genes

The ESTIMATE tool with the R software (version 4.0.5) was used to obtain the proportions of the immune, stromal, and TME components of each CC sample. The results are presented as ImmuneScore, StromalScore, and ESTIMATEScore (16). where a higher score represents a higher proportion. Of the three scores, StromalScore showed the most significant difference in distribution between the GU and GS groups. Differentially expressed genes (DEGs) between the two groups were extracted using the “limma” (17) tool in R, with criteria of false discovery rate (FDR) <0.05 and absolute value of log2 fold change (FC) >1. DEGs from the GU and GS groups were considered as GIRGs, and groups with differently distributed StromalScores were considered stromal-related genes.

### Survival analysis

All survival analyses were done in this paper with the R packages “survival” and “survminer” (18). A total of 230 tumor samples were obtained from 306 patients after excluding patients who met the following criteria (1): days to death ≤1 month (2); normal samples; and (3) incomplete clinical data (19). Results were presented by the survival curve drawn in the Kaplan–Meier method, and a log-rank test with a p-value <0.05 showed statistical significance. Results were presented as survival curves drawn using the Kaplan–Meier method, and a log-rank p-value <0.05 was considered to indicate statistical significance. Then, the effects of gene expression on survival rates were determined using the KM Plotter online database (http://kmplot.com).

### GSEA and functional enrichment analyses

We conducted functional enrichment analyses of the DEGs using the R tools “enrichplot,” “clusterProfiler” (20), and “ggplot2.” Enriched gene ontology terms were identified using the following criteria: p-value <0.05 and q-value <0.05. The C2. CP. KEGG. v7.2, and HALLMARK gene sets were used for the analysis of the different risk cohorts through GSEA, using the GSEA 4.0.3 software. A 5% NOM p-value and a 25% FDR q-value were considered significant.

### Establishment of prognostic model with external verification

As GI and stromal scores were both found to significantly contribute to patient prognosis, the intersection of 947 stromal-related genes and 207 GIRGs was obtained using the R package “VennDiagram.” Next, the 110 intersecting genes were used to construct a prognostic model to predict the survival outcomes of CC patients. The GSE44001 cohort was used to demonstrate the effectiveness of the model as a predictor. The model was constructed as follows: As a first step, we used the “survival” R package to conduct univariate Cox regression analysis with a threshold of p <0.05, and ten key genes were identified for further analyses. Next, LASSO (12) penalized Cox analysis was carried out *via* the R tool “glmnet” (21). Multivariate Cox regression analysis was performed using the “survival” package, then risk scores were exported. The expression data of the four genes were used to calculate risk scores according to the following formula: risk score = expression × coefficient of gene 1 + expression × coefficient of gene 2 + expression × coefficient of gene n (22). The model was verified using the GSE44001 cohort with the same formula. We took the median of the risk scores as a threshold to compartmentalize CC patients into different risk clusters. To assess the predictive effectiveness of the signatures, “survivalROC” (23, 24) was used to calculate the area under the curve, which was proportional to the predictive capability. Other analyses were performed to determine differences in survival between the two groups, such as survival status.

### Analyses of tumor microenvironment and immunotherapy

CIBERSORT (25) and ssGSEA algorithms were used for evaluation of the proportions of tumor-infiltrating cells in all CC samples to determine the differences between the different risk groups. ssGSEA was performed using the “GSVA” R package (26) with an immune gene collection downloaded from MSigDB (https://www.gsea-msigdb.org/). The online tool TIMER (http://timer.cistrome.org/) was employed to explore the relationship between gene expression and immune cells (27). We also investigated how patients in the different risk cohorts responded to immunotherapy. ICPs were obtained from the TISIDB website (http://cis.hku.hk/TISIDB) (28) and already existing studies (29–31). The immunophenoscores of CC samples were obtained from the TCIA (The Cancer Immunome Database; https://tcia.at/home). The IMvigor210 cohort (32) was applied for further validation of the prognostic effect of the hallmarks specific to immunotherapy response. The R packages “limma,” “ggplot,” and “ggplot2” were used to visualize the different distributions of immune components. Information about the immune subtypes of various cancers was obtained from the website UCSC Xena (http://xena.ucsc.edu/).

### Verification experiments

#### Clinical samples

A total of ten cervical cancer tissues were acquired from the Shanghai First Maternity and Infant Hospital, including five early-stage specimens and five late ones. The histological types of acquired samples were all carefully certified as squamous carcinoma by experienced pathologists. The diagnostic standards were based on the latest International Federation of Gynecology and Obstetrics (FIGO) guidelines. All clinical samples have got informed consent with the permission of the Medical Ethics Committee of Shanghai First Maternity and Infant Hospital (Approval notice: KS21264), which were fixed with formalin and embedded with paraffin for further histological analyses.

#### Immunohistochemistry and immunofluorescence of cervical cancer tissues

IHC staining was performed to explore the expression of the RIPOR2 protein in formalin-fixed, paraffin-embedded cervical cancer tissue sections. Primary antibody against RIPOR2 (TD12398, abmart, China) was applied overnight at 4°C. Besides, the sections were further incubated for another 1 h using a secondary antibody (#PK-8501, Vector Lab, USA). Moreover, the Rabbit IgG mini-PLUS Kit was used to detect and visualize the DAB complex (#PK-8501, Vector Lab, USA), along with hematoxylin to counterstain the nuclei. Finally, slides were photographed *via* a microscope. Additionally, IF staining was also conducted using the above sections. After dewaxing and dehydrating, the slides were further treated with tissue microwave antigen retrieval. Then, a blocking procedure was employed at room temperature for 2 h. Afterwards, the slides were added with primary antibody against RIPOR2 (1:150, abclonal, China) overnight at 4°C. After washing the sections three times with PBS for 5 min, Alexa-647 goat anti-rabbit (1:500, CST, America) was applied as the secondary antibody for another 2 h at room temperature. Ultimately, the nuclei were stained with DAPI for 10 min, and the slides were photographed using a confocal microscope.

#### Cell culture, RNA extraction, and real-time qPCR

The SiHa and HeLa CC cell lines were purchased from ATCC (Manassas, VA, USA) for verification experiments. RIPOR2-overexpression plasmids were obtained from the Public Protein/Plasmid Library. The CC cell lines were cultured in high-glucose Dulbecco’s modified Eagle medium (DMEM; Servicebio, China) containing 10% FBS (fetal bovine serum, Biological Industries, Israel) and 1% antibiotic (penicillin/streptomycin; New Cell & Molecular Biotech Co., China) in an incubator at a temperature of 37°C and 5% CO2. The SiHa HeLa cells were then transfected for 48 h with RIPOR2-overexpression plasmids (RIPOR2-pcDNA3.1, 1216 ng/μl) and control empty plasmids (pcDNA3.1, 500 ng/μl) using Lipofectamine 2000 reagent (Invitrogen, USA). RT-qPCR was conducted after transfection to test the efficiency.

We also extracted total RNA from the SiHa and HeLa cells using TRIzol (Invitrogen, USA), measured the concentration and purity of the product, and reversely transcribed it to cDNA with a 5× ALL-IN-One RT Master Mix kit (Applied Biological Materials Inc., Canada). RT-qPCR was performed using a TB Green Premix Ex Taq kit (Takara, Japan), with GAPDH serving as the control. The relative mRNA expression was calculated based on the 2^−△Ct^ method (33). The primers used are shown in **Table S1**.

#### CCK-8 and transwell assays

After successfully transfecting CC cell lines with RIPOR2 (RIPOR2-pcDNA3.1, 1216 ng/μl) and empty (pcDNA3.1) plasmids at a concentration of 500 ng/μl, we used CCK-8 reagent (GeneView, USA) to roughly determine the cell viability. The transfected CC cells were first diluted to a density of 2 × 10^4^ cells/ml; then, 100 μl of cells were plated in each well of 96-well microtiter plates (Coring, NY, USA) for 72 h. Every 24 h, 10 µl of CCK-8 reagent was applied to each well, followed by a further incubation period of 2 h. The optical density (OD) was measured at a wavelength of 450 nm as an indicator of the cell viability. Furthermore, we performed transwell assays to detect the migration ability of the CC cells, with the cell density adjusted to 2 × 10^5^ cells/ml. To establish a suitable concentration gradient to force the cell migration, 150 μl of cell suspension and 800 μl of DMEM containing 20% FBS were distributed, respectively, in the upper and lower chambers of a 24-well plate. At the end of incubation for 24 h at 37°C with 5% CO2, cotton swabs were applied immediately to eliminate the upper-layer cells of the membrane. The upper chamber cells were fixed and dyed with methanol and crystal violet for 15 and 30 min, respectively, and rinsed twice with phosphate-buffered saline. We selected five visual fields at random for imaging and to estimate the quantity of migrated cells.

#### 5−Ethynyl−20−deoxyuridine, cell cycle detection, and colony formation assays

After successfully transfecting CC cells with empty plasmids (pcDNA3.1) and RIPOR2-overexpressing plasmids, we used a BeyoClick™ EdU Cell Proliferation Kit with Alexa Fluor 555 (Beyotime, China) to perform the EdU assay. EdU was added to the cell medium, followed by incubation for 2 h. Afterwards, we used 4% paraformaldehyde to fix the CC cells, followed by permeabilization with 0.3% Triton X-100. The Click solution was then prepared according to the instructions of the manufacturer and used for the incubation of cells for half an hour in the dark. Hoechst 33342 was used to counterstain the cells, and they were imaged and observed *via* an inverted fluorescence microscope (Carl Zeiss, Germany). Cell cycle detection was conducted using the Cell Cycle and Apoptosis Analysis Kit (Beyotime, China). Briefly, PBS and 70% glacial ethanol were used successively to wash and fix the transfected cells at 4 °C for 24 h after CC cells were harvested. Moreover, propidium iodide (PI) was used to suspend the cells and then incubate them for another 30 min in a dark room after washing with PBS. Ultimately, flow cytometry was employed to detect the cell cycle and the frequency of different phases (G0/G1, S, or G2/M) was compared. For the colony formation assay, we seeded CC cells at a density of 800 cells per well in a six-well plate and incubated them for 14 days at the condition of 37°C with 5% CO2. Afterwards, 4% paraformaldehyde and crystal violet were applied to fix and stain the cells, respectively. Colonies were then imaged using a camera and counted.

#### Western blot assay

RIPA buffer supplemented with protease and phosphatase inhibitors (TargetMol, America) was used to lyse the cells. The protein supernatant was then collected and preserved at −80°C. The BCA (Beyotime, China) method was used to detect protein concentrations. The sample proteins were first heated at 99°C for 20 min, then separated by 10% sodium dodecyl sulfate polyacrylamide gel electrophoresis (Servicebio, China) and transferred to polyvinylidene fluoride membranes. After blocking with 5% non-fat milk for 90 min, the membranes were incubated with primary antibodies at 4°C overnight, then washed and incubated with secondary antibodies for another 2 h at room temperature. An enhanced chemiluminescence reagent (Servicebio, China) was used to image the protein bands. The primary antibodies used in this study were PARP1 (1:1,000, CST, America) and GAPDH (1:10,000, Proteintech, USA). The secondary antibodies were purchased from HuaBio, China.

### Statistical analysis

All statistical analyses were performed using the R 4.0.5 software (version 4.0.5), GraphPad Prism 8 (GraphPad, La Jolla, CA, USA), and SPSS 26.0 (IBM, SPSS, Chicago, USA) software. Unpaired Student’s t-tests were used to compare differences between the two groups. P <0.05 served as the cutoff value for a significant difference. All experiments were repeated at least three times.

## Results

### Screening for prognosis-related signatures in CC patients

#### Group division based on GI and stromal score

The GU cohort contained 71 samples, with the number of mutual alterations ranked in the top quarter, while the last quarter (n = 72) in the GS cluster. Using the “limma” R package, we screened out DEG between the GU and GS groups as GIRGs based on the following criteria: |log2 FC| >1, FDR <0.05. A total of 207 GIRGs were obtained for further analysis.

**Figure 1:**
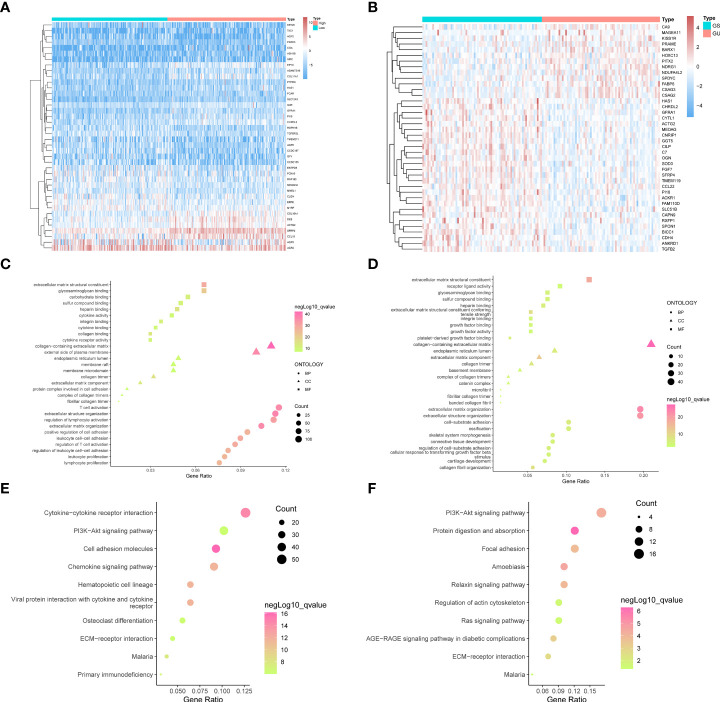
The top forty genes in the StromalScore related genes **(A)** and GIRGs **(B)** groups were respectively selected according to the descending order of the significance of the expression difference. The heatmap was drawn, from the color red to blue, representing the expression from high to low. The result from GO analyses of stromal-related DEGs **(C)** and GIRGs **(D)** was separately displayed. The pathways with the top 10 high gene ratios, respectively, belonging to different oncologies, were shaped into different graphs. In the stromal-related DEGs set, the pathways were mainly enriched in pathways concerning immune cell activity. For GIRGs, there is massive enrichment in pathways related to an extracellular matrix structure. Pathways with the top 10 high gene ratios according to KEGG analyses were exhibited. For stromal-related DEGs **(E)** and GIRGs **(F)**.

CC patients were sorted into GU and GS groups (details in *Group division and other analyses based on mutation data* section), and the correlations of ImmuneScore, StromalScore, and ESTIMATEScore between the two groups were calculated. According to the results, StromalScore showed the most significant difference between the groups, with p = 0.002. Therefore, we divided the CC patients into two groups using the median of the StromalScore as the cut-off. The 947 DEGs between these two groups were filtered out in the same way as the GIRGs, and the top 40 were visualized using heatmaps (**Figures 1A, B**).

**StromalScore appeared to differ most between GU and GS clusters**

Stromal-related DEGs were enriched most in pathways involving immunological-cell activity, for instance, T-cell triggering, adjustment of lymphocyte trends, positive regulation of cellular adhesion, and leukocyte cell–cell adhesion, as well as substance synthesis pathways including carbohydrate binding and glycosaminoglycan binding (**Figure 1C**). GIRGs showed massive enrichment in pathways related to extracellular matrix structure, including extracellular matrix with collagenous components, positive regulation of cell adhesion, organization of extracellular matrix and structure, structure-shaping components, and adhesion between cell and substrate (**Figure 1D**). Kyoto Encyclopedia of Genes and Genomes (KEGG) analysis of stromal-related DEGs yielded pathways with large numbers (more than 30) of enriched genes; these included CCR interaction, chemokine signaling pathway, PI3K–AKT pathway, and cellular adhesion molecules (**Figure 1E**). The results for GIRGs were as follows: PI3K–AKT pathway, the regulation of actin cytoskeleton, protein digestion and absorption, focal cling, amebiasis, relaxin pathway, RAS pathway, AGE–RAGE pathway in diabetic complications, and ECM–receptor interaction (**Figure 1F**).

#### Intersection of stromal-related DEGs and GIRGs considered as prognostic signatures

**Figure 2:**
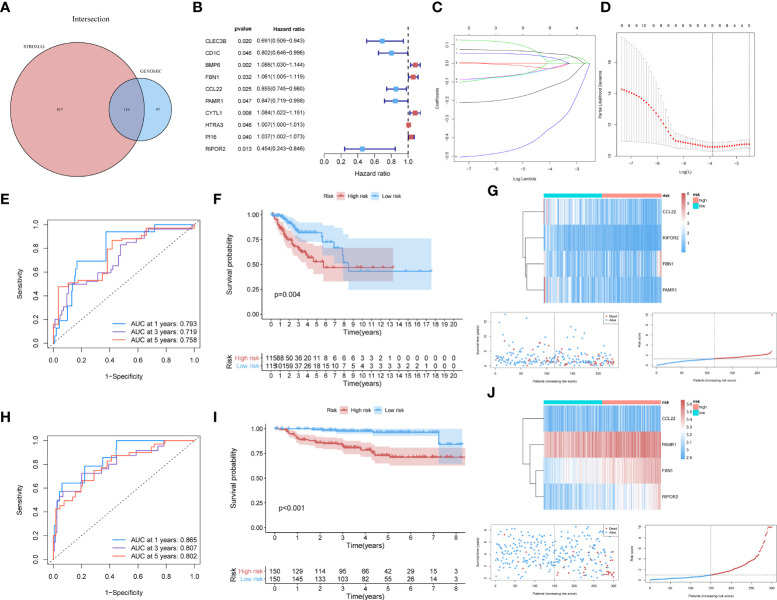
**(A)** The 110 common genes in the intersection analysis of GIRGs and Stromal-Related DEGs. **(B)** 10 prognostic intersection genes filtered out by the univariate Cox analysis with the p-value of <0.05, inclusive of CLEC3B, CD1C, BMP6, FBN1, CCL22, PAMR1, CYTL1, HTRA3, PI16, RIPOR2. **(C, D)** Inferred from the 10-folded LASSO regression results, four signatures were screened out: CCL22, FBN1, RIPOR2, and PAMR1. **(E–G)** Survival distribution-related analyses for the TCGA cohort. **(E)** Survival at 1, 3, and 5 years was drawn in ROC curves. The areas under the curves (AUC) were 0.793 (1 year), 0.719 (3 years), and 0.758 (5 years). **(F)** The result of the Kaplan–Meier analysis showed a higher overall survival rate in the low-risk group with p = 0.004. **(G)** The expression pattern distribution of the model genes was shown by the order of risk scores. **(H–J)** **(H)** For the GEO dataset, the time-ROC went as follows: 0.865 (1 year), 0.807 (3 years), and 0.802 (5 years). **(I)** Survival rate differed with p <0.001. **(J)** The heatmap was also shown.

For constructing a prognostic model, we focused on genes with significant involvement in both GI and TME. Therefore, the intersection of GIRGs and stromal-related DEGs was obtained, resulting in 110 genes for further research (**Figure 2A**).

### Establishment of prognostic model *via* LASSO penalized Cox analysis

#### Model construction

Through univariate Cox analysis using the log-rank test and the “survival” R package, 10 of the 110 intersecting genes were identified as candidates (p <0.05) for LASSO Cox regression [CLEC3B, CD1C, BMP6, fibrillin 1 (FBN1), C–C motif chemokine ligand 22 (CCL22), peptidase domain-containing associated muscle regeneration 1 (PAMR1), CYTL1, HTRA3, PI16, and RIPOR2] (**Figure 2B**). The results are shown in **Table S2**. Details of the LASSO regression and Cox proportional hazard analyses can be found in the *Materials and methods* section. The independent variables (candidate signatures) can be made into a better match and we explored a restrained condition, which was to calculate the | coefficient | and take the summation, for data dimensionality reduction. In the LASSO Cox analysis, we set ‘Cox’ as the family parameter with 10-fold cross-validation for LASSO analysis. The signature genes identified in this analysis were considered covariates and subjected to multivariate Cox analysis. Based on their expression values and regression coefficients, four genes were selected for final model construction: CCL22, FBN1, RIPOR2, and PAMR1 (**Figures 2C, D**). A risk score for each patient was calculated using the following formula: Risk Score = (FBN1 expression ∗ 0.07057) − (CCL22 expression ∗ 0.11517) − (PAMR1 expression ∗ 0.12912) − (RIPOR2 expression ∗ 0.48548).

#### External validation

The data source used for validation was described in the *Establishment of prognostic model with external verification* section. The GSE44001 dataset was processed using the prognostic prediction formula given above. Expression values of the four key genes were set as variables; then, we computed risk scores for each patient based on the GEO data. Patients from the GEO cohort were sorted into different risk cohorts based on the median value of the risk scores, and so were those from the TCGA cohort. During the data processing, various discrepancies were discovered between groups with different risk score distributions. The newly built prognostic risk model still functioned well when using external data for validation. Combined with information about the survival status of CC patients, a series of analyses were conducted in the GEO and TCGA cohorts, and the survival rates were displayed descendingly by the order of risk scores. Receiver operating characteristic curves for 1-, 3-, and 5-year survival of CC patients were constructed to demonstrate the performance of the newly built model. For the TCGA dataset, the area under the curve (AUC) values were 0.793 (1 year), 0.719 (3 years), and 0.758 (5 years) (**Figure 2E**). An area under the curve greater than 0.7 is considered to indicate satisfactory performance of a prognostic model. Kaplan–Meier analyses showed a higher OS rate in the low-risk cluster [p = 0.004 (**Figure 2F**)]. The expression patterns of the signatures in different risk groups were visualized using heatmaps (**Figures 2G**). For the GEO dataset, the AUC values were 0.865 (1 year), 0.807 (3 years), and 0.802 (5 years) (**Figure 2H**). The survival rate differed in high- and low-risk groups, with p <0.001 (**Figure 2I**). These results were also visualized as a heatmap (**Figure 2J**).

### Immune-related and other analyses between different risk cohorts

#### Immune-related cell infiltration analysis, immunotherapeutic response, and GSEA

**Figure 3:**
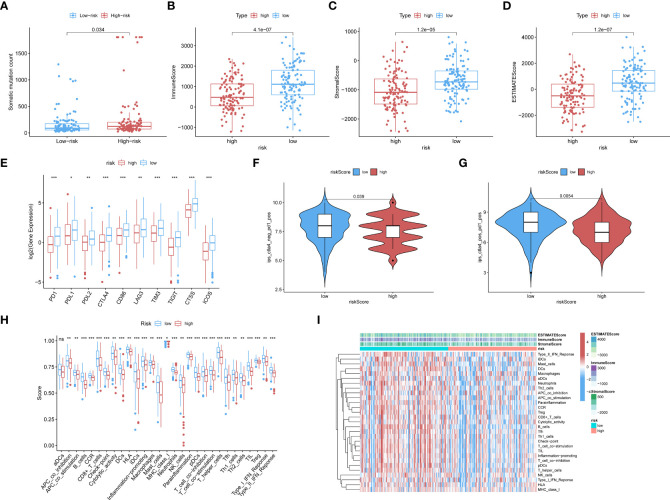
**(A)** The boxplots showed the results of the Wilcoxon test for some characteristic distribution differences between discrepantly-risked clusters, including TMB. The low-risk population takes fewer somatic mutation counts, with a p-value = 0.034. **(B)** Immune Score with p-value = 4.1∗e^−07^, **(C)** Stromal Score with p-value = 1.2∗e^−05^, **(D)** ESTIMATE Score with p-value = 1.2∗e^−07^ and all of them appeared to be higher in low-risk cohort. **(E)** ICPs differently distributed were also depicted in the boxplot, including PD1, PDL1, PD2, CTLA4, CD86, LAG3, TIM3, TIGIT, CTSS, ICOS. (p-value: ***p <0.001, **p <0.01, *p <0.05). **(F, G)** The low-risk cluster had a significantly greater response to immunotherapy of PD1 applied alone with p-value = 0.039 **(F)**, and treatment in combination with CTLA4 and PD1 with p-value = 0.0054 **(G)**. **(H)** According to the ssGSEA calculating results, all the immune cell subpopulations along with immune-related pathway distribution were displayed. **(I)** A corresponding heatmap was shown with an ESTIMATE-related score distribution in the top bars.

The low-risk population generally had lower somatic mutation counts than the high-risk group (p = 0.034) (**Figure 3A**). CC patients in different risk groups also differed with respect to TME-related scores calculated *via* the ESTIMATE algorithm: p = 4.1∗e^−07^ for ImmuneScore (**Figure 3B**), p = 1.2∗e^−05^ for StromalScore (**Figure 3C**), and p = 1.2∗e^−07^ for ESTIMATEScore (**Figure 3D**).

We investigated the associations of risk scores with the expression of ICPs, including PD1, PDL1, PDL2, CTLA4, CD86, and LAG3. The distributions of specific ICPs in different risk cohorts and corresponding p-values are shown in **Figure 3E**. Generally, all the above mentioned ICPs were more highly expressed in the low-risk samples. This applied to all types of ICPs, regardless of whether their role was in immune activation (CD86, ICOS) or immunosuppression (PD1, PDL1, etc.).

Immunophenoscores acquired from the TCIA database were included in the statistics, as shown in the following boxplots; patients with lower risk scores showed significantly greater response to immunotherapy with PD1 alone (p = 0.039; **Figure 3F**) or along with CTLA4 (p = 0.0054; **Figure 3G**).

CIBERSORT and ssGSEA were used to verify the relationship between the model scores and aspects of the TME. We compared the different risk clusters with respect to the distribution of immunological components. Using ssGSEA, we quantified and visualized enrichment scores for various immunological cellular subpopulations, pathways, and functions. As shown in **Figure 3H**, 16 immune cell subpopulations [B cells, CD8+ T cells, macrophages, dendritic cells (DCs), human leukocyte antigen (HLA), mast cells, immature dendritic cells (iDCs), neutrophils, natural killer (NK) cells, T helper cells, plasmacytoid dendritic cells (pDCs), follicular helper T cells, regulatory cells (Tregs), Th1, and Th2 cells, and tumor-infiltrating lymphocytes] and 11 immune-related pathways (type I and type II IFN response, CCR, inflammation promotion, checkpoints, cytolytic activity, parainflammation, APC co-inhibition and co-stimulation, and T-cell co-inhibition and stimulation) had higher scores in the low-risk group compared with the high-risk group (p <0.05). The distributions of immunological components are shown in the heatmap in **Figure 3I** along with the ESTIMATEScore. These findings demonstrate that the risk score based on our model is significantly associated with aspects of the TME, especially immune components.

**Figure 4:**
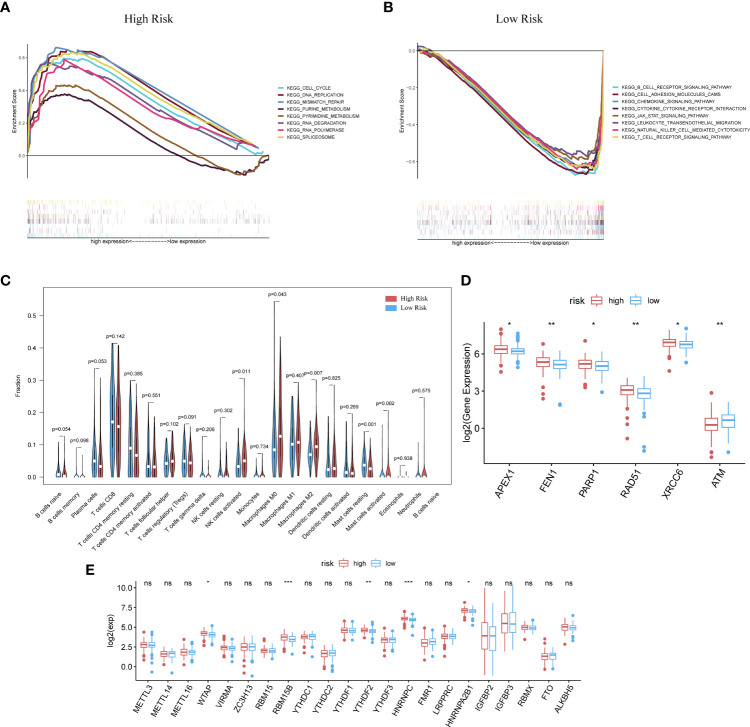
GSEA analysis was done under the high-risk group **(A)** along with the low-risk group **(B)**. **(C)** CIBERSORT analysis result got shown in violin plot. **(D)** Except for ATM, other DDR-related genes with significantly expressed differences are expressed higher in the cluster with high risk (p-Value: ** p < 0.01, * p <0.05). **(E)** M6A methylation-related genes got showed in box-plot (p-Value: *** p < 0.001, ** p < 0.01, * p < 0.05;ns p >0.05).

The GSEA analysis was also used to compare the two risk groups. In the high-risk group, 17 pathways were significantly enriched, of which many were related to GI, for instance, cell cycle, MMR, DNA replication, pyruvate metabolism, and purine metabolism (**Figure 4A**). In contrast, the low-risk group was principally enriched in pathways of immune-related biological functions, including the BCR pathway, chemokine pathway, NK-cell-mediated cytotoxicity, and TCR pathway (**Figure 4B**). The enriched KEGG gene sets are shown in **Table S3**. These results support the ability of the prognostic model to distinguish groups of CC patients at risk of cancer cell proliferation as well as those who have the advantage of an active immune environment.

Analysis with the CIBERSORT algorithm showed a higher proportion of M0 macrophages, natural killer cells, M2 macrophages, and activated mast cells in the high-risk cohort, whereas the low-risk cohort had higher levels of resting mast cells (**Figure 4C**). Some DDR-related genes and m6A methylation-related biomarkers were more enriched in the high-risk group (**Figures 4D, E**).

The scoring system and survival analysis were applied to CC patient groups with different clinical characteristics. The prognostic model was more effective when applied to patients with early-stage CC (clinical and pathological) (**Supplementary Figure 1**).

#### Contrast between high- and low-risk score clusters on somatic mutations

We further investigated how genomic-related features differed between the different risk cohorts. The top 20 samples with the most somatic mutations in each group are shown in **Supplementary Figures 2A, B**. *Via* the “maftools” R package, we used pairwise Fisher’s exact test to detect mutually altered and co-occurring gene sets among the top 20 samples of each group (**Supplementary Figures 2C, D**). MUC17, HUWE1 (HECT, UBA, and WWE domain containing E3 ubiquitin protein ligase 1), DST, ADGRV1, DNAH8, and SPEN were found to have large numbers of mutations only in the high-risk cohort. Fisher’s test was also used to identify differentially mutated genes (DMGs) between the low- and high-risk groups; the results are shown in **Supplementary Figure 2E**. Combining the results of these two analyses, HUWE1, DST, and SPEN were found to mutate more in the high-risk cohort. HUWE1 and EP300 (E1A binding protein P300) showed a positive co-occurrence tendency (p <0.05). The set of samples with mutations in both HUWE1 and EP300 was marked as “Geneset,” and the wild-type samples with no mutations in HUWE1 or EP300 were annotated as “WT.” Then, Kaplan–Meier analysis was performed; the results showed that survival probability differed between mutant and wild types in the high-risk clusters (**Supplementary Figures 2F, G**). In the high-risk group, the wild-type patients showed significantly better survival rates [p = 0.0147; hazard ratio (HR) = 3.47]. However, in the low-risk group, there was no statistically significant difference in survival probability (p = 0.667; HR = 1.09∗e^−07^).

### Further analysis of RIPOR2 as the only ImmuneScore-related model gene

#### Immuno-features and gene function related analyses of hub gene RIPOR2

**Figure 5:**
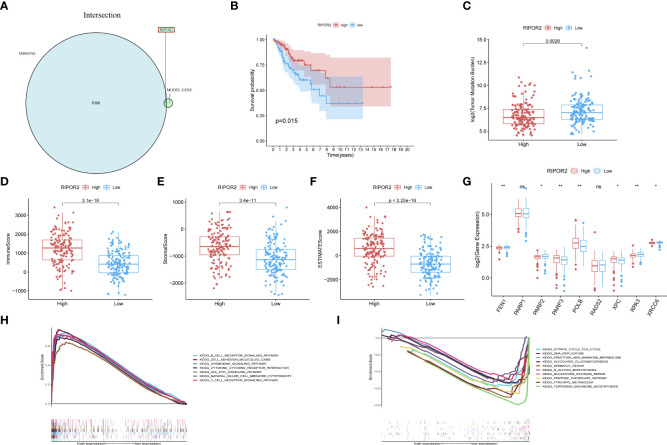
**(A)** RIPOR2 was the only DEG judged by the prognostic model and Immune-Scores. **(B)** Survival analysis for CC patients with different RIPOR2 expression. Patients were marked with high expression or low expression depending on comparing with the median expression level. p = 0.015 by the log-rank test. **(C–F)** The discrepantly distributed features and corresponding p-values were shown in box-plots. The low-RIPOR2 group carried a greater TMB **(C)** with p-value = 0.0028, p-value = 3.1∗e^−16^ **(D)** with Immune Score, p-value = 3.4∗e^−11^ **(E)** with Stromal Score, p-value <2.22∗^−16^ **(F)** with ESTIMATE Score. **(G)** Analysis of DDR-related genes expression showed that FEN1, PARP2, RPA3, XRCC6 expressed more actively in the low-RIPOR2, while PARP3, XPC, ATM went the opposite (p-Value: ** p < 0.01, * p <0.05, ns p > 0.05). **(H, I)** A GSEA analysis was conducted based on the expression level of RIPOR2, with a low-expression cluster **(H)** and a high-expression group **(I)**.

Our previous studies confirmed that ImmuneScore has a significant impact on the prognosis of CC patients (34, 35). Therefore, we selected genes related to the ImmuneScore (GRIS) for further analysis. Setting the median immune score as the cutoff, we divided CC samples into two ImmuneScore-related clusters and followed the same method used for DEG screening above to take the intersection, containing 1,067 genes. So RIPOR2 was targeted and figured out (**Figure 5A**). Patients were sorted into different expression groups based on the median value of RIPOR2, and more bioinformatical analyses were performed, such as immune- and mutation-correlated characteristics and functional enrichment analysis. CC patients with higher RIPOR2 expression were found to have a longer OS time (**Figure 5B**), demonstrating that this gene was a protective factor in CC. The results obtained using the KM Plotter online database also confirmed RIPOR2 as a protective signature in CC patients (**Supplementary Figure 3**).

Generally, the low RIPOR2 expression group carried a greater TMB (**Figure 5C**), as well as lower ESTIMATE-related scores. When we compared the high and low RIPOR2 expression groups using the ESTIMATE algorithm, there were significant differences in ImmuneScore (p = 3.1∗e^−16^; **Figure 5D**), StromalScore (p-value = 3.4∗e^−1^; **Figure 5E**), and ESTIMATEScore (p <2.22∗e^−16^; **Figure 5F**). Moreover, when we analyzed the expression of DDR-related genes in the two groups, as shown in **Figure 5G**, flap structure-specific endonuclease 1 (FEN1), PARP2, RPA3, and XRCC6 were expressed more highly in the low RIPOR2 group, whereas the opposite pattern was seen for PARP3, XPC, and ataxia telangiectasia-mutated (ATM).

A GSEA was conducted in the two RIPOR2 expression groups. Pathways related to mediating fluid immunity and cellular immunity were significantly enriched in the high RIPOR2 expression group; these included the chemokine pathway, cytokine receptor interaction, TCR pathway, BCR signaling pathway, and cellular adhesion molecules (**Figure 5H**). In the low RIPOR2 expression group, most enriched pathways were involved in GI-related biological processes, such as DNA replication and MMR (**Figure 5I**). These above results are the reasons for us to believe that high expression of RIPOR2 promotes the recognition and elimination of tumor cells in the human body *via* processes such as chemotaxis, phagocytosis of immune cells, and processing and presentation of antigens.

**Figure 6:**
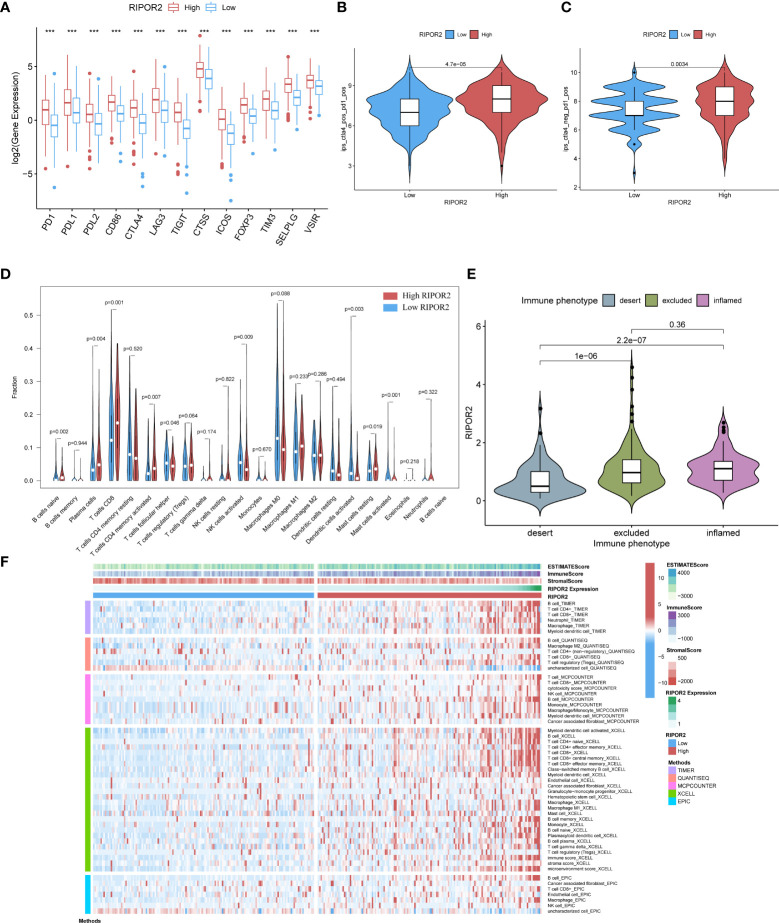
**(A)** The expression of ICPs concerning RIPOR2 was displayed (p-Value: *** p < 0.001). The high expression group responded better under the operation of immunotherapy with PD1 combined with CTLA4 **(B)** or applied alone **(C)**. **(D)** The CIBERSORT result was shown in the comparison between low- and high-RIPOR2 groups by violin plot. **(E)** A statistic on the immune phenotype distribution in different RIPOR2 expression levels comprising “desert,” “excluded,” and “inflamed” were depicted. Patients with low expression of RIPOR2 tend have with the desert phenotype. **(F)** Multialgorithm analytical results on immune cells of tumor microenvironment (TME) in cervical cancer, including existing data from platform TIMER, XCELL, MCP-counter, quanTIseq, and EPIC. The top-bars show the distribution of TME-related scores along with the RIPOR2 expression.

ICPs related to RIPOR2 expression are displayed in **Figure 6A**, the distribution of which confirms the view that RIPOR2 can enhance the immune response. Evidence for this included the better response of the high expression group to immunotherapy with PD1 combined with CTLA4 or alone (**Figures 6B, C**). As one of the most representative immune cells with immuno-functions, CD8+ and activated CD4+ memory T cells showed a significantly higher expression in the high RIPOR2 expression group (**Figure 6D**). The correlations of RIPOR2 expression with CD8+ T and B cells were further explored using the online web tool TIMER; the results showed that RIPOR2 expression had a positive relationship with the above immune cells according to various methods (TIMER, XCELL, MCP-counter, quanTIseq, and EPIC; **Supplementary Figure 4**).

Subsequently, we performed statistical analyses of the immune phenotype distribution in different RIPOR2 expression groups using the IMvigor210 cohort; the phenotypes comprised “desert”, “excluded”, and “inflamed”. Patients with inflamed immune phenotypes commonly show a better response to immunotherapy. As shown in **Figure 6E**, our results provide strong evidence that patients with low expression of RIPOR2 tend to have the desert phenotype. This may limit the benefit they receive from immunotherapy. To further investigate the relationship between RIPOR2 and different immune cells, we applied several bioinformatical methods to determine the correlations (TIMER, XCELL, MCP-counter, quanTIseq, EPIC, etc.). Based on the results of these algorithms, we could conclude that RIPOR2 was strongly related to various immune cells, especially T and B cells (**Figure 6F** and **Table S4**).

#### Pan-cancer analyses about general survival, TME, immuno-subtypes, TMB, MSI, and ICP distributions conducted on RIPOR2

**Figure 7:**
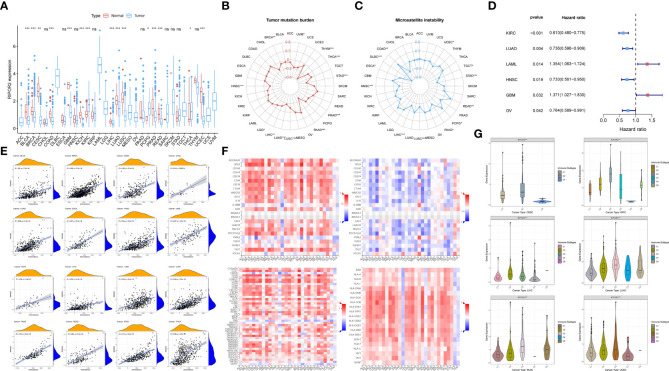
**(A)** The discrepant expression patterns of RIPOR2 in tumorous and normal samples were displayed (p-Value: *** p < 0.001, ** p < 0.01, * p < 0.05, ns p > 0.05). **(B, C)** The radar charts depicted the distributions of TMB **(B)** and microsatellite instability (MSI) **(C)** respectively. Corresponding to the circle representing value zero, the relative position of the points indicates the values of the index. **(D)** The forest plot showed the hazard ratios and p-values of how RIPOR2 affects the prognoses of various tumors. When RIPOR2 functions as a protective indicator, the pot gets drawn in blue when the risky factor is red. **(E)** We showed the results of correlation analyses in dot plots with R and p-values. The orange hill represents the distribution of immune-score and the blue hill of RIPOR2 expression. The positiveness of the relations can be seen from the rising fitting line. **(F)** The grid heatmaps show the relations between RIPOR2 and ICPs with different functions in immune response, inhibitory ICPs on the top-left and the sub-figure on the top-right, in contrast with the association between the expression of methylated RIPOR2 distribution and the same ICPs. The stimulatory ICP group is in the bottom-left corner, and HLA-related genes are on the bottom-right. **(G)** The samples can be divided into six immune subtypes: C1–C6 corresponding to Wound Healing, IFN-γ Dominant, Inflammatory, Lymphocyte Depleted, Immunologically Quiet, and TGF-β Dominant. The scattering situation was shown in boxplots. If any of the immune subtypes do not exist in a cancer species, they will not appear in the sub-picture.

To determine whether the protective role of RIPOR2 was unique to CC or also existed in other cancers, we conducted a pan-cancer analysis of RIPOR2 expression levels using data from the TCGA. This was also intended as preparation for further research on the underlying principles of the model. The discrepant expression patterns of RIPOR2 in tumor and normal samples are displayed in **Figure 7A**. Based on a comprehensive analysis of 33 cancer types, we concluded that RIPOR2 was generally a protective factor in tumor patients. Nine cancer types showed no significant differences between normal and tumor tissues, and nine types for which no normal sample data were available for comparison. However, RIPOR2 was expressed at significantly higher levels in normal tissues than tumor tissues in patients with bladder urothelial carcinoma, breast invasive carcinoma, CC, colon adenocarcinoma (COAD), glioblastoma (GBM), kidney chromophobe, liver hepatocellular carcinoma (LIHC), lung adenocarcinoma (LUAD), lung squamous cell carcinoma, prostate adenocarcinoma, rectum adenocarcinoma (READ), and uterine corpus endometrial carcinoma (UCEC). In contrast, kidney renal clear cell carcinoma (4), pheochromocytoma and paraganglioma (PCPG), and thyroid carcinoma showed higher expression in tumor samples compared with normal tissues.

**Figures 7B, C** depict the distributions of TMB and microsatellite instability (MSI), respectively. In the pan-cancer analyses, high RIPOR2 expression was significantly associated with lower TMB in all cancer types, as well as with lower MSI in most tumor types except COAD, PCPG, and READ. The results of the univariate Cox proportional hazard regression analysis showed that the expression of RIPOR2 was associated with the OS of cancer patients. It served as a protective factor in KIRC, LUAD, head and neck squamous cell carcinoma, and ovarian serous cystadenocarcinoma, and as a risk factor in acute myeloid leukemia and GBM (**Figure 7D**).

As mentioned earlier, RIPOR2 expression was correlated with immune scores calculated *via* ESTIMATE in CC. When we explored potential interactions between RIPOR2 expression and immune scores on a larger scale, a highly positive relationship was discovered in all tumor types. The strongest correlation was found for pancreatic adenocarcinoma (PAAD; R = 0.79, p <2.2e^−16^) (**Figure 7E**).

Thereafter, we analyzed ICPs to determine the influence of RIPOR2 on the immune environment of tumor patients. ICP molecules include both inhibitory and stimulatory types; therefore, they have diverse effects on the tumor immune microenvironment (TIME). The above results suggest that both types of ICPs are expressed in numerous tumor types. Inhibitory ICPs have long been considered to mediate immune evasion in tumor patients; they also function in many other malignant behaviors, including self-renewal, metastasis, and drug resistance. As shown in **Figure 7F**, most of the 24 inhibitory ICPs showed positive correlations with RIPOR2 in most of the 30 cancer types considered here, with the exceptions of brain lower grade glioma (LGG), GBM, PCPG, uterine carcinosarcoma (UCS), and uveal melanoma (UVM). Compared with the results obtained with the same cancer types and methylated RIPOR2 on the top-right, the color block shows the corresponding degree of negative correlation, consistent with the previous results. However, when the same analysis was applied to the 45 stimulatory ICP genes, RIPOR2 had a close correlation with the expression of ICPs. With the exceptions of LGG, PCPG, and UVM, strong correlations between RIPOR2 and ICP expression were found in all cancer types. Human leucocyte antigen (HLA), the product of the major histocompatibility complex in humans, is in charge of induction and regulation of the immune response. HLA-related genes were found to correlate with RIPOR2 expression in the pan-cancer analysis in all cancers apart from LGG, PCPG, UCS, and UVM. In conclusion, RIPOR2 was shown to have universal relevance in the triggering of immune responses in multiple cancers.

Based on immune subtype theory, patients across cancer types can be identified into six immunological subtypes denoted C1–C6, corresponding to wound healing, IFN-γ dominant, inflammatory, lymphocyte depleted, immunologically quiet, and TGF-β dominant (36). We analyzed cancer types for which immune-subtype information was available from the UCSC Xena database. According to the results, CC, KIRC, LIHC, PAAD, LUAD, and UCEC patients with the C2 and C3 subtypes—that is, a hotter tumor immune microenvironment and a better response to immunotherapy—had the highest levels of RIPOR2 expression (**Figure 7G**).

### Validated experiments for RIPOR2 in CC

#### IHC and immunofluorescence demonstrated RIPOR2 expressed differently in different stages

**Figure 8:**
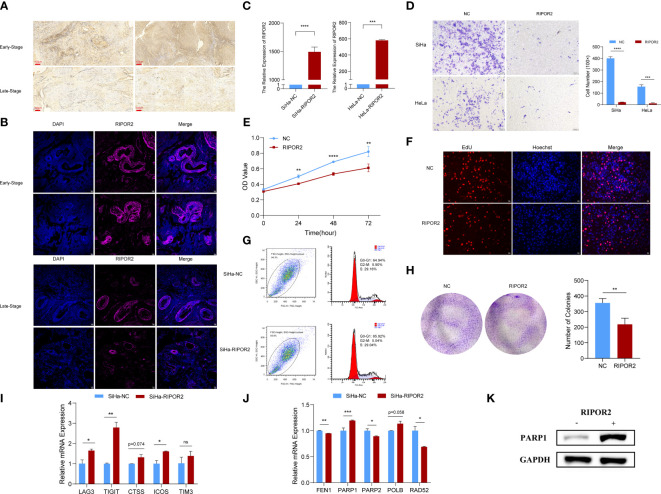
Functional experimental verification of RIPOR2. **(A)** The expression of RIPOR2 protein in early- and late-stage cervical cancer tissues was detected by Immunohistochemistry (IHC), Scale bar, 100 μm. **(B)** Immunofluorescence **(I, F)** staining results of RIPOR2 protein in early- and late-stage of cervical cancer tissues. Scale bar, 25 μm. **(C)** The expressing amount of RIPOR2 differed in the control cell lines and overexpression transfected cell lines, both in the SiHa group with p-value <0.0001(****) and the HeLa group with a p-value <0.001(***). **(D)** The results of the transwell assay, pictures on the left show the CC cell distributions of groups photographed in different visual fields. “NC” means normal control with pcDNA3.1 (empty vector) transfection; “RIPOR2” represents a group with overexpression plasmid transfection. The result of differential analysis based on cell number was shown in the bar plot. **(E)** It is shown that at times of 24, 48, and 72 h, the HeLa cells living in the normal control group are always significantly greater than the RIPOR2-overexpressed group by CCK-8 (p-value: **p <0.01, ****p <0.0001). **(F)** EdU assay was used to detect the proliferation ability of each group of SiHa cells. Scale bar, 100 μm. **(G)** Flow cytometry analysis showed overexpression of RIPOR2 in SiHa cells slightly increased the percentage of the G0/G1 phase cells and decreased the percentage of S and G2/M phage cells. **(H)** Colon formation assay was performed to determine the proliferation ability of each group of cells. The bar plot showed the number of colonies in SiHa cells, with p <0.01 (**). **(I)** Inferred from RT-qPCR, checkpoints LAG3, TIGIT, CTSS, ICOS, and TIM3 showed correspondingly high expression in the SiHa cell lines transfected with RIPOR2-overexpressing plasmids. **(J)** RT-qPCR results indicated that FEN1, PARP2, and RAD52 negatively correlated with RIPOR2, PARP1 expressed more in the SiHa-RIPOR2 group, and POLB got a higher expression amount in the RIPOR2 group however with p-value = 0.058. **(K)** “−” represents the normal control group, while “+”is for the RIPOR2-overexpressed cluster. The protein level of PARP1 is much stronger with RIPOR2-overexpression of SiHa cells, which corresponds to the results of the RNA level.

Clinical specimens of cervical cancer patients at different pathological stages were acquired to further investigate the expression of RIPOR2 protein using IHC and immunofluorescence experiments. The results demonstrated that the RIPOR2 expression was higher in samples with early stage than in late stage (**Figures 8A, B**), which illustrated that RIPOR2 expression was decreasing along with advanced stage classification.

#### Transwell, CCK-8, EdU, cell cycle detection, and colony formation assays indicated RIPOR2 as an anti-tumor signature

RIPOR2 was successfully overexpressed in SiHa cells with p <0.0001(****) and HeLa cells with p <0.001 (***) using transfection with plasmids as described in the *Verification experiments* section (**Figure 8C**). Compared with normal control CC cells, the RIPOR2-overexpression group showed significantly weaker migration ability, as demonstrated by transwell assay; this was the case for both SiHa and HeLa cells (**Figure 8D**). Additionally, according to the results of the CCK-8 assay, the viability of HeLa cells was markedly suppressed by RIPOR2 transfection as inferred from the OD values (**Figure 8E**). Moreover, the results of EdU assays further confirmed that RIPOR2 overexpression could inhibit the proliferation of SiHa CC cells compared with the control group (**Figure 8F**). Besides, the results of cell cycle detection revealed that overexpressing RIPOR2 in SiHa cells could slightly increase the percentage of G0/G1 phase cells and decrease the proportion of S and G2/M phase cells (**Figure 8G**), although this association was not statistically significant. The colony formation assay also demonstrated that RIPOR2 could significantly inhibit the proliferation of SiHa cells (**Figure 8H**).

#### Relationship between RIPOR2 and DDR-related genes and ICPs

As mentioned in the *Verification experiments* and *Transwell, CCK-8, EdU, cell cycle detection, and colony formation assays indicated RIPOR2 as an anti-tumor signature* sections, the correlation of RIPOR2 with ICPs was further verified by experiments. Checkpoints LAG3, TIGIT, CTSS, ICOS, and TIM3 showed high expression in SiHa cells transfected with RIPOR2-overexpression plasmids (**Figure 8I**). Through RT-qPCR, we demonstrated experimentally that FEN1, PARP2, and RAD52 were negatively correlated with RIPOR2 expression; PARP1 was expressed more highly in the RIPOR2-overexpression group; and POLB had the same correlation trend with PARP1, although the difference was non-significant (p = 0.058) (**Figure 8J**). PARP1 was an important factor related to DDR, and its correlation with RIPOR2 at the protein level was further verified by the WB assay in SiHa cells. It could be clearly seen that the PARP1 protein level was much higher in the RIPOR2-overexpression group (**Figure 8K**).

## Discussion

According to the results of our bioinformatic analyses, the low survival rate of the high-risk group identified by our model is accompanied by high mutation loads, high expression of DDR-related genes (FEN1, PARP1, RAD51, ATM, etc.), and poorer response to PD-1-based immunotherapy.

GI plays a dual role in cancer treatment and is a hallmark of DDR-deficient cancer. As they accumulate, mutations may accelerate the evolution of cancer cells; moreover, deleterious mutations can also create vulnerabilities in cancer cells (37). In addition to GI, the TME plays an important part in the main malignant tumors affecting women (including epithelial ovarian and endometrial cancers as well as CC). Owing to the strong crosstalk between stromal and cancer cells, the environment in which they co-exist develops viciously circulative properties (38). Evidence from the previously published pieces of literature shows that the TME may interact with GI, potentially *via* DDR. The TME may decrease genomic stability, *via* inhibition of DDR pathways. DDR defects help the immune system with tumor cell recognition, resulting in greater exposure of DDR-deficient cancer cells to the adaptive immune system (39–41).

Among the differentially expressed DDR-related genes, ATM and FEN1 cooperate with intracellular reactive oxygen species to regulate RhoA activation, which contributes to cell survival (42) as well as being involved in DDR (43, 44). The recruitment of PARP1 is essential to all stages of DDR processes in various types of DNA lesions. As PARP1 is an abundant nuclear protein, a negatively charged polymer adheres to it after translation, and this poly-ribosylation activity supports the repair of single-strand and double-strand breaks, and well as most of the known functions of PARP1 in the DDR pathway (45–47). The RAD51 gene in the Homologous Recombination Repair (HRR) pathway is related not only to DDR but also to hypoxia; thus, it is indirectly connected to the increase in GI (41). As so many DDR-related key factors showed significantly increased expression in high-risk samples according to our results, it is reasonable to speculate that the significantly more frequent occurrence of DDR and consequent acceleration of immune invasion in cancer cells is among the reasons for the higher risk level of this patient group.

To further investigate the underlying principles of our prognostic risk model, we considered the function of the prognostic candidates in a tumorous environment. FBN1, a risk factor according to our analysis, has been shown by previous research to be positively correlated with carcinogenesis, as well as with a higher risk of tumor development in a pan-cancer analysis based on a million-case Taiwan cohort (48). CCL22, a protein secreted by M2 macrophages, recruits Th2 cells, resulting in better survival (48). The gathering effect seems to work on Treg cells as well; however, it shows a contrary influence in CC (49, 50). Consistent with our results, PAMR1 has long been considered an inhibitor of tumor activities such as spread, migration, and development in CC. In CC tissues, PAMR1 was expressed at lower levels than in normal samples and was associated with worse survival. Evidence suggests that PAMR1 participates in the suppression of MYC targets and mTORC1 signaling pathways (51).

As mentioned above, the TME plays a vital role in the effect exerted by GI on the survival of tumor patients. Here, we focused on RIPOR2, as the only gene in the intersection between the GIRG and GRIS groups. RIPOR2, a member of the recently discovered RIPOR (RHO family interacting cell polarization regulator) family, exerts an inhibitory influence on RHO activity and on cellular functions affected by RHO. RIPORs were previously reported to play a role in cell polarization and migration, owing to phosphorylation induced by chemokine stimulation, which influences their interplay with RHO and has consequent effects on the activation of RHO downstream targets. In most tumor types, RIPOR2 expression is relatively suppressed compared with normal samples, particularly in gynecologic malignancies like endometrial carcinoma. Evidence to date suggests that RIPOR2 is a pan-cancer protective factor. However, the precise role of RIPOR2 in carcinogenesis and tumor development remains to be clarified, and there are still pressing questions left to be explored.

Of note, in addition to its role as a downregulator of RHOA, RIPOR2 functions in cellular proliferation independently of RHOA *via* its inhibitory regulation of HDAC6. HDAC6, a cytoplasmic deacetylase that accelerates tumor proliferation by triggering deacetylase activity, can bind to phosphorylated RIPOR2 and 14-3-3, forming a tripartite complex and disrupting its original function of forming a mitotic spindle. However, RIPOR2 with a S5A mutation lacks this antiproliferative function. Another impressive finding is the inner relationship between RIPOR2 and T cells. In our analyses of immune cell expression in this study, the patient group with high expression of RIPOR2 had significantly higher levels of CD8+ T cells. Previous research suggests that RIPOR2 has a RHOA-dependent impact on various T-cell activities, including polarization, clinging, and migration of T cells (52).

The differences in survival between different CC patient groups according to risk level may be related to immune checkpoint inhibitors (ICIs). Inspired by newly identified biomarkers and neoantigens created by DDR alterations, DDR-targeting drugs have emerged as a promising therapeutic approach. These drugs may function better in combination with ICI-based immunotherapies, according to experimental studies demonstrating the successful application of PD-1 suppressors in cancers with MMR deficiency (53). Combination therapy is of particular use in situations where insufficient neoantigens are produced to activate the immune system. The interaction between immunoregulators and DDR factors may improve the stimulation of the immune response against tumor cells (54).

As mentioned before, PARP1 is an essential participant in the DDR process. Owing to its functions in promoting cell stability and survival, PARP1 is considered to contribute to the immune evasion and drug resistance of cancer cells. Therefore, PARP1 inhibitors are widely used for anti-cancer treatment. PARP1 has been explored to influence immune components in some types of cancers (55, 56), which may also explain the higher proportion of inflamed immune subtypes in low-risk CC patient populations. However, the application of PARP inhibitors has its limitations. For example, blockade of PARP1 inactivates glycogen synthase kinase 3β, resulting in dose-dependent upregulation of PD-L1 and thus suppressing T-cell activity. Combinations of PD-L1 blockers are tailored to address this situation by restoring the sensitivity of tumor cells to the T-cell response. Combining PARP inhibitors with anti-PD-L1 antibodies (alazaparib, olparib, and rucaparib) in therapeutic modalities resulted in a better response than either drug alone (57).

The repair and maintenance of PARP1 also affects immune cells. In the process of lymphatic proliferation, coordinated signals from PARP1 and PARP2 are needed to maintain genomic integrity, for instance, in the maturation of T cells (58, 59). Either PARP1 or PARP2 deficiency alone does not affect the quantity of T cells; however, a dual deficiency will lead to a significant reduction in numbers of peripheral blood T cells such as CD4+ and CD8+ T cells (59). In mouse experiments, decreased numbers of B cells were observed in the bone marrow of animals with dual PARP1 and PARP2 gene defects. Similar to the case of T cells, this was caused by an accumulation of unrepaired DNA damage during proliferation (14, 60). PARP1 and PARP2 also participate in the natural immune response, including the activities of phagocytes and NK cells. It is worth noting that PARP1 is essential for the inflammatory response of phagocytes; for instance, in macrophages, it accelerates the transcription of pro-inflammatory genes and saves M1 macrophages from death under oxidative conditions (61, 62). Recent studies have shown that PARP1 makes an essential contribution to NK cell biology, for instance, in gathering NK cells to a virus-infected area and participating in killing tumor cells (63–65). In this study, RIPOR2 was found to possess a strong relationship with PARP1 expression at both the RNA and protein levels; thus, it could serve as a meaningful signature for use in decision-making about the clinical treatment of CC patients.

This comprehensive analysis indicates that the difference in survival between different risk score groups is related to interactions among GI, the stromal environment, and DDR-related genes and the influence of these factors on the effectiveness of immunotherapy. Among the various factors identified here, the core protective gene RIPOR2 in the model made the greatest contribution to many aspects, such as survival and immunotherapy response. Thus, the expression of RIPOR2 is a potential effective marker in CC patients, with applications in predicting response to immunotherapy.

## Conclusion

To sum up, we successfully constructed a four-signature CC prognostic risk model based on GI and TME related factors. The model has been verified to predict all-stage CC patients effectively. The efficacy of prediction may result from the comprehensive interaction between GI, DDR, and TME. RIPOR2 showed its potential ability to foreshadow prognosis, its positive relationship with immunological functioning cells like T cells and ICPs, and a promisingly better response to immunotherapy when highly expressed. Comprehensive bioinformatical analyses combined with corresponding validated experiments have indicated that RIPOR2 was a protective factor in CC, which also appeared to have a close relationship with immunotherapy responses. Furthermore, RIPOR2 has also shown links with diversely functioning ICPs in plenty of cancer types, the summation of which tends to enhance immunity. In all, RIPOR2 can be taken as a promising signature, predicting better prognosis with a wide scope of application.

## Data availability statement

The original contributions presented in the study are included in the article/**Supplementary Material**. Further inquiries can be directed to the corresponding authors.

## Ethics statement

The studies involving human participants were reviewed and approved by Medical Ethics Committee of Shanghai First Maternity and Infant Hospital. The patients/participants provided their written informed consent to participate in this study.

## Author contributions

SX, SL, and QH conceived and designed the manuscript. FX, CZ, and YG processed and annotated the data. FX and CZ cooperated to accomplish the validating experiments. CZ and YG composed the manuscript. JS and TL proofread the manuscript along with data review. All authors contributed to the article and approved the submitted version.

## Funding

This work was supported by the Shanghai Science and Technology Planning Project (grant no. 21Y11907000), the Natural Science Foundation of Shanghai (grant no. 21ZR1450900), the National Natural Science Foundation of China (grant no. 81772762), and the Clinical Science and Technology Innovation Project of Shanghai Shenkang Hospital Development Center (grant no. SHDC12019X34).

## Conflict of interest

The authors declare that the research was conducted in the absence of any commercial or financial relationships that could be construed as a potential conflict of interest.

## Publisher’s note

All claims expressed in this article are solely those of the authors and do not necessarily represent those of their affiliated organizations, or those of the publisher, the editors and the reviewers. Any product that may be evaluated in this article, or claim that may be made by its manufacturer, is not guaranteed or endorsed by the publisher.
