# Supplementary material for: Comprehensive analyses identify RIPOR2 as a genomic instability-associated immune prognostic biomarker in cervical cancer
Source: Front Immunol. 2022 Aug 26;13:930488. doi: 10.3389/fimmu.2022.930488 (PMC9458976; doi:10.3389/fimmu.2022.930488)
Supplement: Supplementary file 1 [file DataSheet_1.pdf]

# Supplementary Material

## 1.1 Supplementary Tables

**Table S1.** Primer nucleotide sequence of this study.

| Gene | Primer nucleotide sequence |
|---|---|
| GAPDH | Forward: 5’-CTGGGCTACACTGAGCACC-3’ |
| | Reverse: 5’-AAGTGGTCGTTGAGGGCAATG-3’ |
| RIPOR2 | Forward: 5’- GAAAATTCCTCCGCTCTCAAGA-3’ |
| | Reverse: 5’- TTCAAGGCCCTGTAGACTTCT-3’ |
| FEN1 | Forward: 5’-ATGACATCAAGAGCTACTTTGGC-3’ |
| | Reverse: 5’-GGCGAACAGCAATCAGGAACT-3’ |
| PARP1 | Forward: 5’- CGGAGTCTTCGGATAAGCTCT-3’ |
| | Reverse: 5’- TTTCCATCAAACATGGGCGAC-3’ |
| PARP2 | Forward: 5’- GCCTTGCTGTTAAAGGGCAAA-3’ |
| | Reverse: 5’- TCCTTCACAATACACATGAGCC-3’ |
| POLB | Forward: 5’-TGGAAAAGATTCGGCAGGATG-3’ |
| | Reverse: 5’-CAGATGGACCAATGCCACTAAC-3’ |
| RAD52 | Forward: 5’- CCAGAAGGTGTGCTACATTGAG-3’ |
| | Reverse: 5’- ACAGACTCCCACGTAGAACTTG-3’ |
| TIGIT | Forward: 5’- TCTGCATCTATCACACCTACCC-3’ |
| | Reverse: 5’- CCACCACGATGACTGCTGT-3’ |
| CTSS | Forward: 5’-TGTAGATGCGCGTCATCCTTC-3’ |

| Gene | Primer nucleotide sequence |
|---|---|
| | Reverse: 5’-CCAACCACAAGTACACCATGAT-3’ |
| ICOS | Forward: 5’- CAGGAGAAATCAATGGTTCTGCC-3’ |
| | Reverse: 5’- CCTTTTGTCTTAGTGAGATCGCA-3’ |

**Table S2.** The univariate-cox results of differentially expressed genes.

| id | HR | HR.95L | HR.95H | pvalue |
|---|---|---|---|---|
| CLEC3B | 0.690849 | 0.506049 | 0.943135 | 0.019881 |
| CD1C | 0.802257 | 0.646196 | 0.996007 | 0.04591 |
| BMP6 | 1.085786 | 1.030463 | 1.144079 | 0.002038 |
| FBN1 | 1.060564 | 1.005184 | 1.118995 | 0.03164 |
| CCL22 | 0.85454 | 0.744968 | 0.980228 | 0.024756 |
| PAMR1 | 0.847191 | 0.719052 | 0.998164 | 0.047488 |
| CYTL1 | 1.084405 | 1.021689 | 1.150972 | 0.007679 |
| HTRA3 | 1.006744 | 1.000122 | 1.013409 | 0.045898 |
| PI16 | 1.036754 | 1.001712 | 1.073021 | 0.039641 |
| RIPOR2 | 0.453699 | 0.243171 | 0.846493 | 0.013003 |

**Table S3.** Enriched KEGG gene sets of different risk groups.

| Description | ES | NES | p-value | FDR | group |
|---|---|---|---|---|---|
| KEGG_CELL_CYCLE | 0.59674 | 1.92415 | 0.00201 | 0.06038 | High risk |
| KEGG_SPLICEOSOME | 0.63125 | 1.91733 | 0.00606 | 0.03233 | High risk |
| KEGG_N_GLYCAN_BIOSYNTHESIS | 0.64276 | 1.86973 | 0.00791 | 0.04056 | High risk |
| KEGG_PROTEIN_EXPORT | 0.70734 | 1.82669 | 0.00401 | 0.05685 | High risk |
| KEGG_RNA_DEGRADATION | 0.57288 | 1.81678 | 0.00389 | 0.05098 | High risk |
| KEGG_AMINOACYL_TRNA_BIOSYNTHESIS | 0.64241 | 1.77450 | 0.01040 | 0.06488 | High risk |
| KEGG_TERPENOID_BACKBONE_BIOSYNTHESIS | 0.71830 | 1.77232 | 0.00411 | 0.05626 | High risk |
| KEGG_MISMATCH_REPAIR | 0.66328 | 1.70069 | 0.01569 | 0.09526 | High risk |
| KEGG_PYRUVATE_METABOLISM | 0.51599 | 1.68877 | 0.01237 | 0.09301 | High risk |
| KEGG_GLYCOLYSIS_GLUCONEOGENESIS | 0.50413 | 1.68764 | 0.00815 | 0.08399 | High risk |
| KEGG_RNA_POLYMERASE | 0.58910 | 1.62400 | 0.02806 | 0.12781 | High risk |
| KEGG_DNA_REPLICATION | 0.64999 | 1.61066 | 0.03400 | 0.13123 | High risk |
| KEGG_HOMOLOGOUS_RECOMBIN | 0.56954 | 1.57700 | 0.04038 | 0.15620 | High risk |

| Description | ES | NES | p-value | FDR | group |
|---|---|---|---|---|---|
| ATION | | | | | |
| KEGG_PENTOSE_PHOSPHATE_PATHWAY | 0.53921 | 1.55018 | 0.04225 | 0.17324 | High risk |
| KEGG_FRUCTOSE_AND_MANNOSE_METABOLISM | 0.48456 | 1.53295 | 0.03482 | 0.17885 | High risk |
| KEGG_PYRIMIDINE_METABOLISM | 0.43214 | 1.53061 | 0.04356 | 0.17052 | High risk |
| KEGG_NUCLEOTIDE_EXCISION_REPAIR | 0.50213 | 1.52218 | 0.07115 | 0.16836 | High risk |
| KEGG_ONE_CARBON_POOL_BY_FOLATE | 0.57612 | 1.51231 | 0.05182 | 0.16867 | High risk |
| KEGG_BASE_EXCISION_REPAIR | 0.51368 | 1.50909 | 0.06346 | 0.16299 | High risk |
| KEGG_GLYOXYLATE_AND_DICARBOXYLATE_METABOLISM | 0.57387 | 1.50820 | 0.07143 | 0.15585 | High risk |
| KEGG_PURINE_METABOLISM | 0.37741 | 1.49544 | 0.02713 | 0.16024 | High risk |
| KEGG_BIOSYNTHESIS_OF_UNSATURATED_FATTY_ACIDS | 0.47879 | 1.42048 | 0.08964 | 0.23451 | High risk |
| KEGG_CYTOKINE_CYTOKINE_RECEPTOR_INTERACTION | -0.63866 | -2.26796 | 0.00000 | 0.00000 | Low risk |
| KEGG_CELL_ADHESION_MOLECULES_CAMS | -0.67478 | -2.25669 | 0.00000 | 0.00000 | Low risk |
| KEGG_B_CELL_RECEPTOR_SIGNALING_PATHWAY | -0.68066 | -2.23792 | 0.00000 | 0.00000 | Low risk |
| KEGG_CHEMOKINE_SIGNALING_PATHWAY | -0.63126 | -2.22908 | 0.00000 | 0.00000 | Low risk |
| KEGG_HEMATOPOIETIC_CELL_LINEAGE | -0.74466 | -2.19673 | 0.00000 | 0.00026 | Low risk |
| KEGG_JAK_STAT_SIGNALING_PATHWAY | -0.59643 | -2.17246 | 0.00000 | 0.00021 | Low risk |
| KEGG_NATURAL_KILLER_CELL_MEDIATED_CYTOTOXICITY | -0.64697 | -2.14504 | 0.00000 | 0.00049 | Low risk |
| KEGG_T_CELL_RECEPTOR_SIGNALING_PATHWAY | -0.65157 | -2.12726 | 0.00000 | 0.00043 | Low risk |
| KEGG_FC_EPSILON_RI_SIGNALING_PATHWAY | -0.57696 | -2.11629 | 0.00000 | 0.00062 | Low risk |
| KEGG_LEISHMANIA_INFECTION | -0.70381 | -2.10595 | 0.00000 | 0.00065 | Low risk |
| KEGG_AUTOIMMUNE_THYROID_DISEASE | -0.78279 | -2.09016 | 0.00000 | 0.00059 | Low risk |
| KEGG_PRIMARY_IMMUNODEFICIENCY | -0.86387 | -2.09002 | 0.00000 | 0.00054 | Low risk |
| KEGG_LEUKOCYTE_TRANSENDOTHELIAL_MIGRATION | -0.58167 | -2.05034 | 0.00000 | 0.00121 | Low risk |
| KEGG_INTESTINAL_IMMUNE_NETWORK_FOR_IGA_PRODUCTION | -0.79264 | -2.01267 | 0.00000 | 0.00180 | Low risk |
| KEGG_TOLL_LIKE_RECEPTOR_SIGNALING_PATHWAY | -0.57815 | -2.00354 | 0.00000 | 0.00196 | Low risk |
| KEGG_VIRAL_MYOCARDITIS | -0.63201 | -1.94945 | 0.00412 | 0.00380 | Low risk |

| Description | ES | NES | p-value | FDR | group |
|---|---|---|---|---|---|
| KEGG_ASTHMA | -0.76574 | -1.91912 | 0.00996 | 0.00584 | Low risk |
| KEGG_FC_GAMMA_R_MEDIATED_PHAGOCYTOSIS | -0.53958 | -1.91324 | 0.00000 | 0.00595 | Low risk |
| KEGG_ABC_TRANSPORTERS | -0.58830 | -1.91302 | 0.00204 | 0.00564 | Low risk |
| KEGG_EPITHELIAL_CELL_SIGNALING_IN_HELICOBACTER_PYLORI_INFECTION | -0.53917 | -1.87432 | 0.00000 | 0.00888 | Low risk |
| KEGG_DORSO_VENTRAL_AXIS_FORMATION | -0.61904 | -1.84740 | 0.00202 | 0.01152 | Low risk |
| KEGG_ALLOGRAFT_REJECTION | -0.81949 | -1.84207 | 0.00609 | 0.01172 | Low risk |
| KEGG_VEGF_SIGNALING_PATHWAY | -0.48438 | -1.83552 | 0.00000 | 0.01191 | Low risk |
| KEGG_ACUTE_MYELOID_LEUKEMIA | -0.54467 | -1.82763 | 0.00410 | 0.01252 | Low risk |
| KEGG_PHOSPHATIDYLINOSITOL_SIGNALING_SYSTEM | -0.53848 | -1.81898 | 0.00000 | 0.01345 | Low risk |
| KEGG_COMPLEMENT_AND_COAGULATION_CASCADES | -0.58616 | -1.79560 | 0.00409 | 0.01746 | Low risk |
| KEGG_RIG_I_LIKE_RECEPTOR_SIGNALING_PATHWAY | -0.51033 | -1.76365 | 0.00428 | 0.02433 | Low risk |

**Table S4.** Correlation of RIPOR2 with different immune cells via different methods.

| Immune Cells | pvalue |
|---|---|
| B cell_TIMER | 8.07E-16 |
| T cell CD4+_TIMER | 1.63E-13 |
| T cell CD8+_TIMER | 1.85E-10 |
| Neutrophil_TIMER | 1.42E-08 |
| Macrophage_TIMER | 0.000506363 |
| Myeloid dendritic cell_TIMER | 3.05E-14 |
| B cell_QUANTISEQ | 8.29E-11 |
| Macrophage M2_QUANTISEQ | 3.52E-09 |
| T cell CD4+ (non-regulatory)_QUANTISEQ | 0.007591646 |
| T cell CD8+_QUANTISEQ | 5.57E-09 |
| T cell regulatory (Tregs)_QUANTISEQ | 1.08E-13 |
| uncharacterized cell_QUANTISEQ | 1.88E-12 |
| T cell_MCPCOUNTER | 1.07E-13 |
| T cell CD8+_MCPCOUNTER | 1.11E-06 |
| cytotoxicity score_MCPCOUNTER | 5.39E-08 |
| NK cell_MCPCOUNTER | 5.47E-08 |
| B cell_MCPCOUNTER | 2.25E-19 |
| Monocyte_MCPCOUNTER | 9.63E-08 |
| Macrophage/Monocyte_MCPCOUNTER | 9.63E-08 |
| Myeloid dendritic cell_MCPCOUNTER | 6.02E-11 |
| Cancer associated fibroblast_MCPCOUNTER | 0.0082272 |

| Immune Cells | pvalue |
|---|---|
| Myeloid dendritic cell activated_XCELL | 4.61E-13 |
| B cell_XCELL | 2.40E-14 |
| T cell CD4+ naive_XCELL | 1.36E-10 |
| T cell CD4+ effector memory_XCELL | 0.000196619 |
| T cell CD8+_XCELL | 2.27E-13 |
| T cell CD8+ central memory_XCELL | 2.36E-14 |
| T cell CD8+ effector memory_XCELL | 6.76E-07 |
| Class-switched memory B cell_XCELL | 1.51E-07 |
| Myeloid dendritic cell_XCELL | 4.11E-12 |
| Endothelial cell_XCELL | 0.000364991 |
| Cancer associated fibroblast_XCELL | 1.80E-05 |
| Granulocyte-monocyte progenitor_XCELL | 0.00400137 |
| Hematopoietic stem cell_XCELL | 0.005322691 |
| Macrophage_XCELL | 0.000125615 |
| Macrophage M1_XCELL | 2.70E-06 |
| Mast cell_XCELL | 0.020239227 |
| B cell memory_XCELL | 3.29E-10 |
| Monocyte_XCELL | 0.000812046 |
| B cell naive_XCELL | 7.11E-06 |
| Plasmacytoid dendritic cell_XCELL | 7.37E-07 |
| B cell plasma_XCELL | 5.70E-06 |
| T cell gamma delta_XCELL | 0.000117466 |
| T cell regulatory (Tregs)_XCELL | 0.036740797 |
| immune score_XCELL | 2.08E-16 |
| stroma score_XCELL | 1.41E-05 |
| microenvironment score_XCELL | 3.53E-19 |
| B cell_EPIC | 2.16E-17 |
| Cancer associated fibroblast_EPIC | 0.017421554 |
| T cell CD8+_EPIC | 0.002618484 |
| Endothelial cell_EPIC | 4.20E-05 |
| Macrophage_EPIC | 1.57E-08 |
| NK cell_EPIC | 5.58E-05 |
| uncharacterized cell_EPIC | 2.61E-07 |

## 1.2 Supplementary Figures

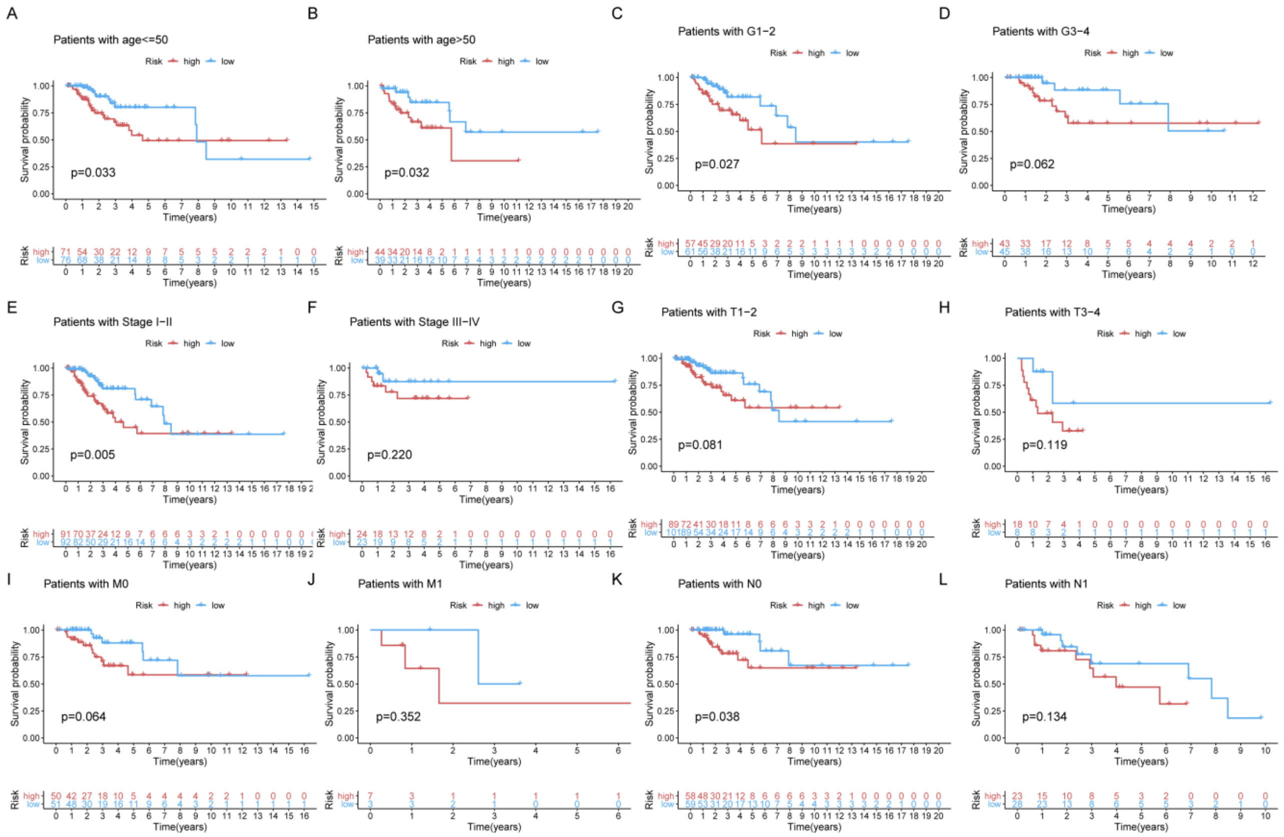


**Supplementary Figure 1. Kaplan-Meier curves of overall survival (OS) for cervical cancer patients' clinicopathological characteristics based on risk score.** (A, B) Kaplan-Meier curves of OS in age <= 50 and age > 50 CC. (C, D) Kaplan-Meier curves of OS in G1-2 and G3-4. (E, F) Kaplan-Meier curves of OS in Stage I-II and Stage III-IV. (G, H) Kaplan-Meier curves of OS in T1-2 and T3-4. (I, J) Kaplan-Meier curves of OS in M0 and M1. (K, L) Kaplan-Meier curves of OS in N0 and N1.

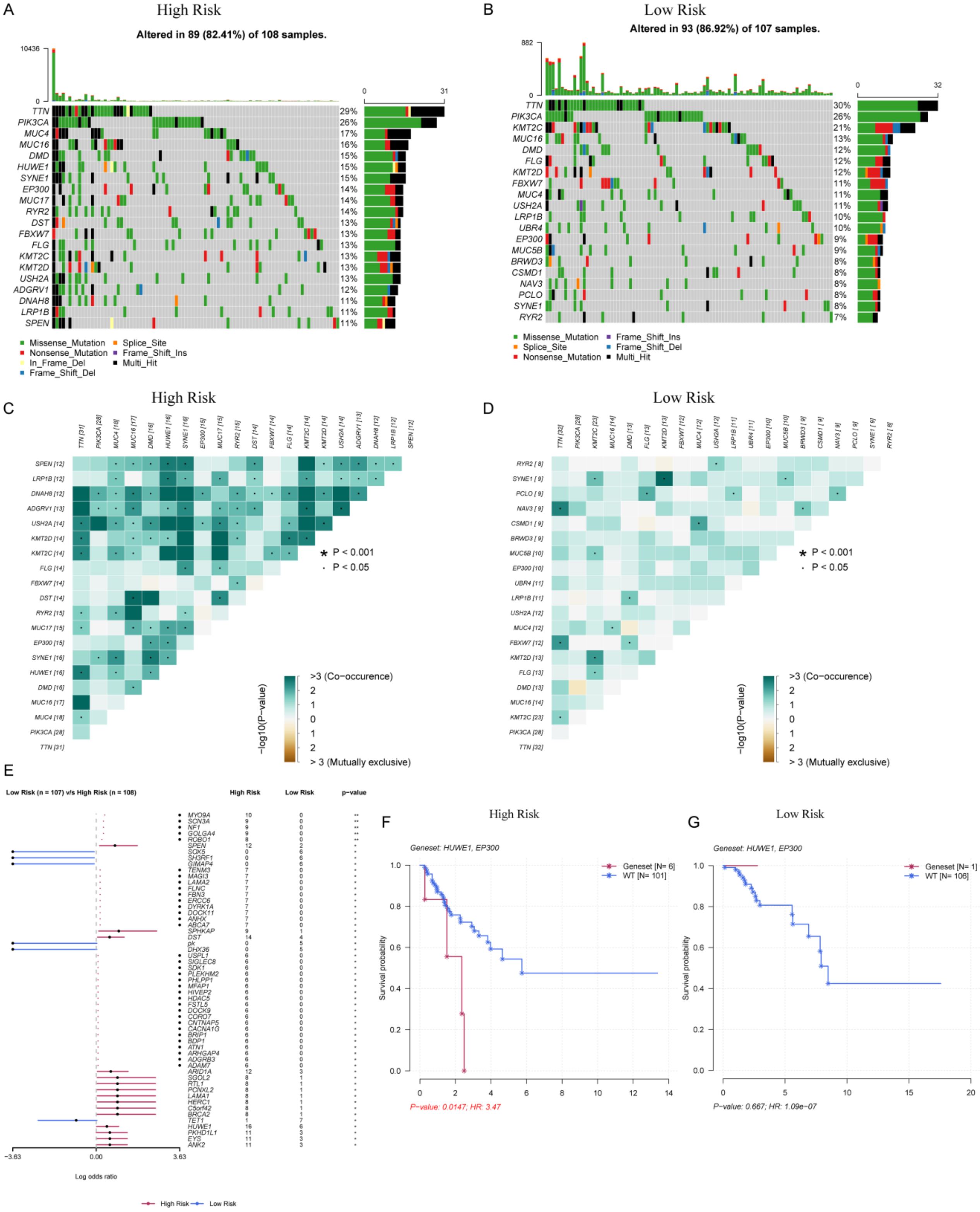


**Supplementary Figure 2. Landscape of Somatic Mutation in High-Risk and Low-Risk Cohorts.** (A, B) Waterfall plot shows the mutation distribution of the top 20 most frequently mutated genes. The

central panel shows the types of mutations in each cervical cancer (CC) sample. The upper panel shows the mutation frequency of each CC sample. The bar plots on the left and right side show the frequency and mutation type of genes mutated in the low-risk and high-risk cohort, respectively. The bottom panel is the legend for mutation types. (C, D) The heatmap illustrates the mutually co-occurring and exclusive mutations of the top 20 frequently mutated genes. The color and symbol in each cell represent the statistical significance of the exclusivity or co-occurrence for each pair of genes. (E) Forest plot displays the significantly differentially mutated genes between two cohorts. (F, G) Kaplan-Meier curves show the independent relevance between overall survival time and HUWE1 and EP300 mutation in high-risk and low-risk cohorts.

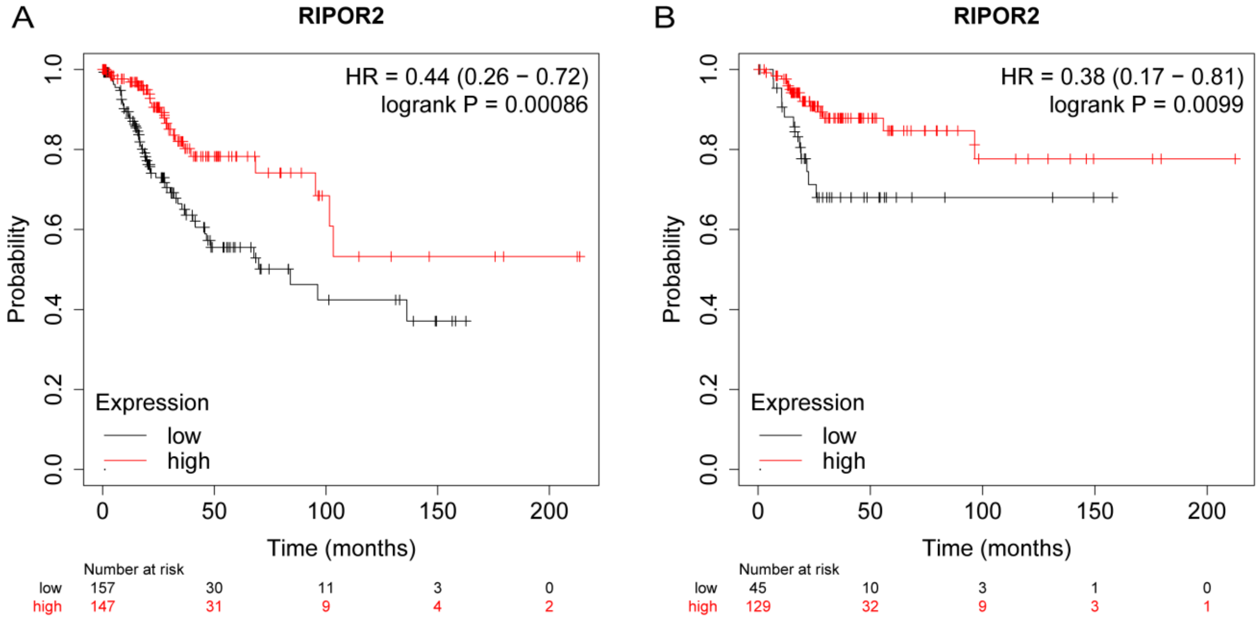


**Supplementary Figure 3. The correlation of APOBEC3A expression with survival time using the Kaplan-Meier plotter.** (A) Overall Survival (OS) analysis for CC patients with different RIPOR2 expression by log-rank test, p = 0.00086. (B) Relapse free survival (RFS) analysis for CC patients with different RIPOR2 expression by log-rank test, p = 0.0099.

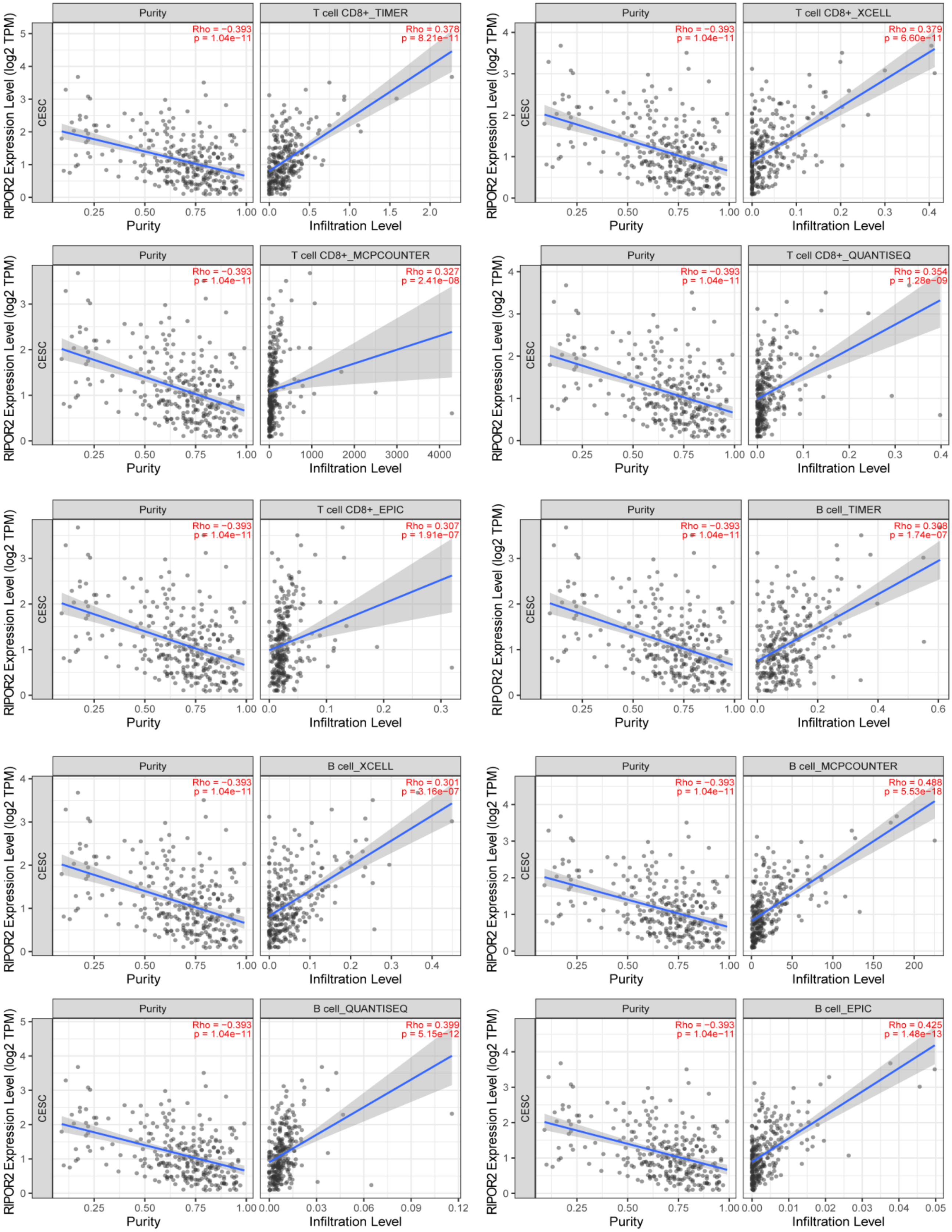
Purity
T cell CD8+_TIMER
Rho = −0.393
p = 1.04e−11
Rho = 0.378
p = 8.21e−11
Purity
T cell CD8+_XCELL
Rho = 0.379
p = 6.60e−11
T cell CD8+_MCPCOUNTER
Rho = 0.327
p = 2.41e−08
T cell CD8+_QUANTISEQ
Rho = 0.354
p = 1.28e−09
T cell CD8+_EPIC
Rho = 0.307
p = 1.91e−07
B cell_TIMER
Rho = 0.308
p = 1.74e−07
B cell_XCELL
Rho = 0.301
p = 3.16e−07
B cell_MCPCOUNTER
Rho = 0.488
p = 5.53e−18
B cell_QUANTISEQ
Rho = 0.399
p = 5.15e−12
B cell_EPIC
Rho = 0.425
p = 1.48e−13
CESC
RIPOR2 Expression Level (log2 TPM)
Infiltration Level

**Supplementary Figure 4. Correlation of RIPOR2 expression with immune cells via TIMER online database.**
